# A study of degradation mechanisms in PVDF-based photovoltaic backsheets

**DOI:** 10.1038/s41598-022-18477-1

**Published:** 2022-08-24

**Authors:** Soňa Uličná, Michael Owen-Bellini, Stephanie L. Moffitt, Archana Sinha, Jared Tracy, Kaushik Roy-Choudhury, David C. Miller, Peter Hacke, Laura T. Schelhas

**Affiliations:** 1 SLAC National Accelerator Laboratory, Menlo Park, CA USA; 2 National Renewable Energy Laboratory, Golden, CO USA; 3 DuPont Specialty Products LLC, Wilmington, DE USA

**Keywords:** Photovoltaics, Characterization and analytical techniques

## Abstract

Commercial backsheets based on polyvinylidene fluoride (PVDF) can experience premature field failures in the form of outer layer cracking. This work seeks to provide a better understanding of the changes in material properties that lead to crack formation and find appropriate accelerated tests to replicate them. The PVDF-based backsheet outer layer can have a different structure and composition, and is often blended with a poly(methyl methacrylate) (PMMA) polymer. We observed depletion of PMMA upon aging with sequential (MAST) and combined (C-AST) accelerated stress testing. In field-aged samples from Arizona and India, where PVDF crystallizes in its predominant α-phase, the degree of crystallinity greatly increased. MAST and C-AST protocols were, to some extent, able to replicate the increase in crystallinity seen in PVDF after ~ 7 years in the field, but no single-stress test condition (UV, damp heat, thermal cycling) resulted in significant changes in the material properties. The MAST regimen used here was too extreme to produce realistic degradation, but the test was useful in discovering weaknesses of the particular PVDF-based outer layer structure studied. No excessive β-phase formation was observed after aging with any test condition; however, the presence of β-phase was identified locally by Fourier transform infrared spectroscopy (FTIR). We conclude that both MAST and C-AST are relevant tests for screening outdoor failure mechanisms in PVDF backsheets, as they were successful in producing material degradation that led to cracking.

## Introduction

**Figure 1:**
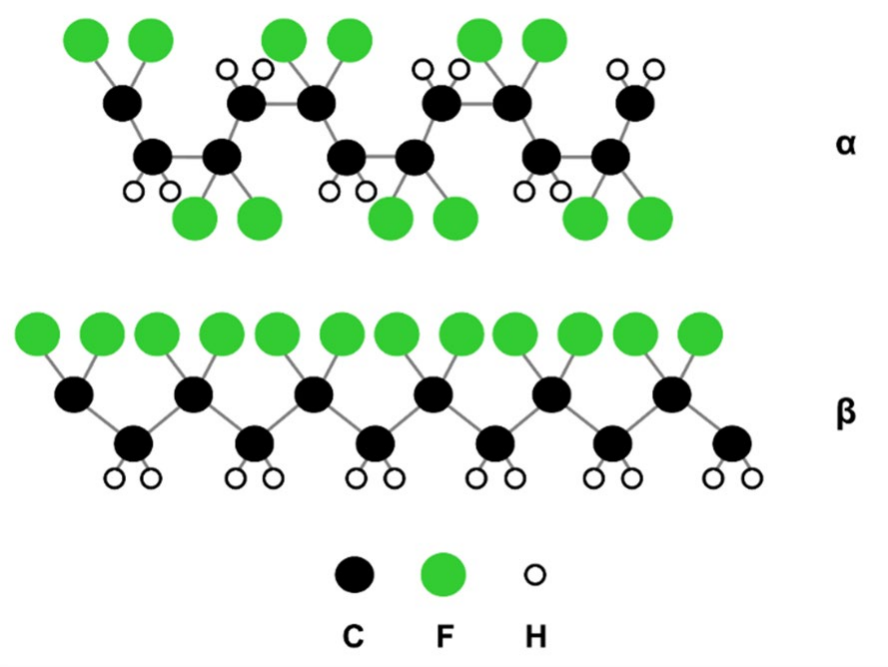
Schematic of PVDF α- and β-phase molecular chain conformation.

Backsheets constitute the rear side outermost layer of protection for the active components of standard photovoltaic (PV) modules. One typical backsheet type is comprised of an opaque multi-layer laminated polymeric sheet on the rear side of the module. A thicker core layer provides insulating properties and mechanical strength. Thinner inner and outer layers are designed for good adhesion to the encapsulant and resistance to the outdoor environment. Polyethylene terephthalate (PET) is a popular choice for the core layer, and fluoropolymers, such as polyvinyl fluoride (PVF) and polyvinylidene fluoride (PVDF), are commonly used for the inner/outer backsheet layers. In this work, we will focus on the PVDF-based backsheet structure (i.e., backsheets containing at least one layer of PVDF polymer). PVDF-based backsheets currently make up ~ 50% of the world market share^1^. PVDF is a semi-crystalline thermoplastic fluoropolymer formed of covalent C–H and C–F bonds. PVDF has high purity, excellent chemical inertness, mechanical abrasion resistance, and UV stability^2,3^. High electronegativity and dissociation energy of the C–F bond ensure good thermal stability of the polymer^4^. As is common in many polymers, PVDF can have different molecular chain conformations, i.e., orientation of alternating –CF_2_– and –CH_2_– units. When C–F dipoles are oriented in the same direction (trans-planar zig-zag conformation, TTTT), the polymer is in its crystalline PVDF β-phase. In the case of antiparallel C–F dipole packing, the polymer is in its non-polar α-phase (TGTG’ conformation)^5–7^. PVDF α- and β-chain conformations are visualized in a schematic in Fig. 1. The α-phase is the most common phase, as it can be obtained by crystallization from melt. The β-phase can be formed by mechanical deformation via uniaxial or biaxial drawing of the α-phase below 100 °C^6^. Under special conditions, PVDF can form other polymorphs (γ, δ, and ε), but these are less common^8^.

To further complicate our understanding of the polymers in backsheets, the PVDF outer layer is a complex material containing pigments and additives, and it is typically blended with acrylic polymers as well [e.g., poly(methyl methacrylate) (PMMA)]^9–12^. Depending on the manufacturer, fabrication process, and composition, the layer may have different physical and mechanical properties. PVDF-based backsheets in deployed PV modules have been seen to fail prematurely. Failure here is defined as cracking. Backsheet cracking can not only compromise the module operating power by enabling enhanced ingress of moisture and oxygen, but it also presents an electrical hazard by exposing the high-voltage components. In a recent field study conducted by DuPont, 23% of investigated PVDF-containing modules were defective by year 9 of deployment^13^. Cracks were seen to form along busbars; however, no clear correlation with a particular climate was found.

The mechanisms behind the observed PVDF-based backsheet field cracking are not fully understood. Single-stress test conditions, such as the 85 °C and 85% relative humidity (RH) damp heat (DH) test, UV exposure, and thermal cycling (TC), produce material degradation, but have not been successful at replicating failures observed in the field. This is likely due to a lack of synergistic effects^14–16^. In recent years, more sophisticated test protocols have been designed to incorporate some of the complexities of outdoor environments. DuPont’s Module Accelerated Stress Test (MAST) sequentially applies DH, UV, and TC. One cycle of MAST was demonstrated to produce overt microcracking in some PVDF-based backsheet films in the machine direction (i.e., the direction parallel to the extrusion, with polymer chains oriented longitudinally)^15^. However, different types of crack formation were observed when the MAST sequence was modified by omitting the UV exposure, with a single crack formed near the junction box^17^. Combined-accelerated stress testing (C-AST) developed at NREL successfully reproduced field failures in commercial backsheets, including PVDF^18,19^. In the early stages of development, C-AST combined “tropical” and “multi-seasonal” protocols to simulate different climates. Cracks appeared on the PVDF-based backsheet over outward-facing topographic features when a long tropical sequence was followed by just one week of the “desert” test sequence in the multi-seasonal protocol. The desert condition applies mechanical load and TC at low RH. Under further aging in the desert sequence, cracks spread rapidly over the entire backsheet^19^.

To understand how cracking formed in aged PVDF-based backsheets, Moffitt et al. studied microstructural changes in PVDF/PMMA backsheet outer surface using atomic force microscopy (AFM), and scanning electron microscopy (SEM). Pits and voids from the loss of PMMA and TiO_2_ particles were observed upon aging. Mechanical stretching of the aged polymer formed micro-cracks by connecting these pits. In addition, a different polymer ordering was observed near the crack tip, which suggests a possible local phase change due to higher level of strain^20^. This work seeks to understand the mechanisms behind PVDF failure with respect to the test conditions applied. Material properties, including chemical composition, degree of crystallinity, and phase formation, are investigated. PVDF-based backsheets that underwent single-stress, sequential, and combined-accelerated testing are compared to failed fielded PVDF-based backsheets from two different climates.

## Experimental procedures

We applied various stress-test conditions to PVDF-based backsheets incorporated into silicon PV modules with poly(ethylene co-vinyl acetate) (EVA) encapsulant or as laminated glass/EVA/backsheet coupons. The single-stress test conditions included 2000 h of DH with a chamber temperature of 85 °C and 85% RH per IEC 61215-2 test protocol^21^; 1500 h of UV exposure (65 W/m^2^ at 340 nm, chamber temperature of 60 °C); and 200 thermal cycles (TCs) between − 40 and 85 °C^21^. The sequential stress test (MAST) applied the following in sequence: (1) 1000 h of DH (85 °C/85% RH), (2) 1000 h of UV-A (65 W/m^2^ between 300 and 400 nm at 70 °C black panel temperature), (3) three rounds of the following: 200 TCs (− 40 to 85 °C) followed by 1000 h of UV-A^15^. C-AST aging consisted of two protocols: a tropical protocol combining DH, UV, humidity freeze, mechanical loading, system voltage, and rain spray to simulate hot and humid climates, and a multi-seasonal protocol with low humidity, TC, and mechanical load to simulate continental climates. Details of the C-AST protocols’ test conditions can be found elsewhere^19^. A summary table of all accelerated stress test conditions applied can be found in Supplementary Table S1 in the Supplementary Information (SI). Backsheets from the fielded modules were exposed to an arid climate in Arizona, USA, for 7 years (7y AZ), and to a hot, semi-arid climate with seasonal cooling in western India for 7.5 years (7.5y India).

Two types of opaque (white-pigmented) PVDF-based backsheet layers were used in this study. A single-layer PVDF/PMMA blend with a thickness of 25 μm (PVDF-A) was used in single-stress test conditions, MAST, and C-AST aging. A co-extruded three-layer structure composed of PVDF|(PVDF/PMMA)|PVDF with a total thickness of 25 μm (PVDF-B) was used in the fielded modules, MAST, and single-stress tests. Because of the limited chamber space and available test schedule, only PVDF-A backsheet was tested using C-AST. To the best information and diagnosis in this study, the three-layer PVDF-B is believed to be the same structure, composition, and fabricated by the same process in the reference sample as in the field aged samples, however a change in processing of the PVDF-B cannot be absolutely ruled out. Table 1 summarizes the sample types and stress test conditions applied.

**Table 1:** Backsheet samples and stress-test conditions.

		
Unaged		X	X
Single	DH	X	X
	UV	X	X
	TC	X	X
Sequential	MAST	X	X
Combined	C-AST	X	
Fielded	7 years, Arizona		X
	7.5 years, India		X

The PVDF-based backsheets examined in this study were extracted from PV modules (fielded modules), mini-modules (C-AST and MAST), and laminated coupons (single-stress tests and unaged). Backsheets were cut from the modules, and subsequently, the PVDF-based outer layer was separated from the PET core by soaking the backsheets in acetone to dissolve the interlayer adhesive. This approach has been used previously without signs of damage to the polymers^22,23^. Only the PVDF-based outer layer (type A and B) was used for material characterization, in order to focus on the PVDF material degradation. An unaged PVDF-based layer from a laminated glass/EVA/backsheet coupon was characterized along with the stressed samples to serve as a reference.

The PVDF-based outer backsheet layers were analyzed to detect changes in physical, chemical, and structural properties after various stress tests. All samples in Table 1 were characterized using the three techniques described here. Differential scanning calorimetry (DSC) was performed using a TA Instruments Q2000 equipped with a RCS90 refrigerating unit to detect polymer thermal transitions between − 90 and 250 °C. Circular backsheet samples of ~ 6 mg were crimped in a non-hermetic aluminum pan and subjected to a heat-cool-heat cycle under flowing nitrogen at the rate of 50 mL/min, with a heating/cooling rate of 10 °C/min. Fourier transform infrared spectra (FTIR) in attenuated total reflection (ATR) mode were collected using a Bruker Alpha II with a platinum ATR attachment and diamond crystal. For each measurement, 75 scans were performed with a measurement resolution of 2 cm^−1^. The spectra were normalized to the C–F stretching band at 1180 cm^−1^, which is common to both α- and β-PVDF phases. The C–F bond is not expected to change with aging. Wide-angle X-ray scattering (WAXS) diffractograms were obtained at Stanford Synchrotron Radiation Lightsource beamline 11-3 at SLAC National Accelerator Laboratory. Rectangular backsheet samples were exposed in transmission geometry to the X-ray beam of energy 12.7 keV (0.9744 Å) for 30 s at a sample-to-detector distance of 200 mm (CCD area detector Rayonix MX225). The data was calibrated using a lanthanum hexaboride (LaB_6_) standard and analyzed using the GSAS-II software package^24^. 1-D diffraction patterns as a function of scattering vector $Q=\frac{4\pi }{\lambda }\mathrm{sin}(\frac{2\theta }{2})$ were obtained by integrating 2-D scattering data. Igor Pro’s (WaveMetrics Inc.) multi-peak fitting package was used to deconvolute WAXS and FTIR spectra fitting Gaussian peaks. A single spectrum for each sample was fitted and the fitting algorithm estimates an error of the fit. Due to the challenge of fitting the complex background in the WAXS data, the amplitudes of peaks corresponding to the α- and β-phases were compared instead of the peak areas.

## Results and discussion

### Effect of aging on PMMA in the PVDF/PMMA blend (PVDF-A)

As noted previously, backsheet polymers are not monolithic, but rather are composites of different polymers and additives. To reduce cost and improve adhesion properties, PVDF is often blended with an amorphous acrylic polymer, PMMA. PMMA has good insulating properties, chemical resistance, high rigidity, and good miscibility with PVDF^3,25^. Typical PMMA content is 20–40 wt%; however, the exact PVDF to PMMA ratio, as well as the additive content of commercial PVDF, is often proprietary. In this study, the degradation of two different PVDF-based backsheet layers is investigated. One type contains PMMA on the weathering-exposed surface (PVDF-A), whereas the other type has a layered structure with no PMMA at the surface (PVDF-B). FTIR is a surface-sensitive technique that allows detection of chemical changes in the outermost 2 μm of the backsheet exposed surface. Unlike additives, PMMA is easily distinguished from the PVDF FTIR spectra by the presence of the carbonyl (C=O) peak located at 1730 cm^−1^. Changes in the peak intensity can indicate degradation related to the PMMA component in the backsheet.

**Figure 2:**
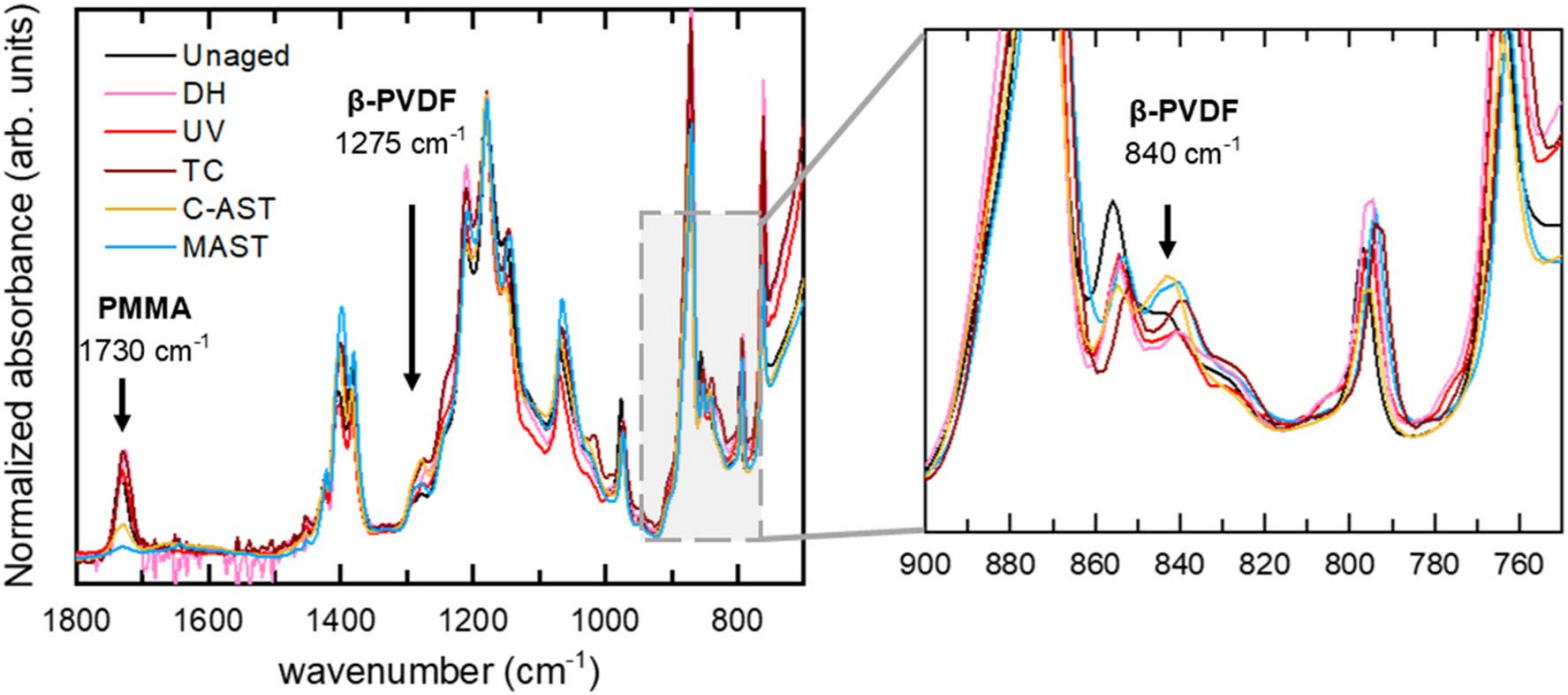
Normalized FTIR spectra of the weathering-exposed surface of the single-layer PVDF-A. Labelled peaks are specific to the PMMA polymer and β-PVDF phase; other sharp unlabeled peaks are from PVDF peaks common to all phases or exclusive to α-PVDF. The inset on the right shows the detail in the region of 900–750 cm^−1^ containing the second β-PVDF FTIR peak.

Figure 2 shows the FTIR spectra of the single-layer PVDF-A on the unaged sample and after the stress tests. The FTIR spectra contain sharp peaks that predominantly correspond to the α-PVDF crystal phase and the carbonyl peak from the PMMA in the blend^26^. The intensity of the PMMA peak was greatly reduced after C-AST aging, and the peak nearly disappeared after the MAST regimen. This is indicative of PMMA depletion on the exposed surface of the backsheet. PVDF is known for high resistance to UV, although Gu et al*.* showed that UV exposure can cause acrylic degradation, leading to mass loss in the form of gaseous products^27^. Miller et al. showed that PMMA is vulnerable to chain scission from UV and/or elevated temperature, and observed the formation of pores and cracks related to the mass loss at the local scission site^28^. No reduction of the PMMA carbonyl peak was observed after any of the single-stress test conditions, suggesting that the sequential and combined accelerated tests had a greater effect on the stability of the PVDF-A blend. No PMMA can be detected by FTIR in the PVDF-B type backsheet, as the outermost PVDF layer is thicker than the penetration depth of the measurement (see Supplementary Fig. S1 in the SI). Because the PVDF-B is co-extruded, the PVDF/PMMA core layer cannot be extracted and analyzed directly.

Varying the content of PMMA relative to PVDF has previously been shown to induce changes to the polymer’s degree of crystallinity, crystalline phase, and melting and crystallization temperature, due to a thermodynamic effect formed in a crystalline-amorphous polymer mixture^10,25^. Here, DSC is used to evaluate changes in the crystallinity and transition temperatures of the PVDF-based backsheet layer. Unlike FTIR, DSC is not surface sensitive; it reflects changes throughout the entire PVDF-based layer thickness.

**Figure 3:**
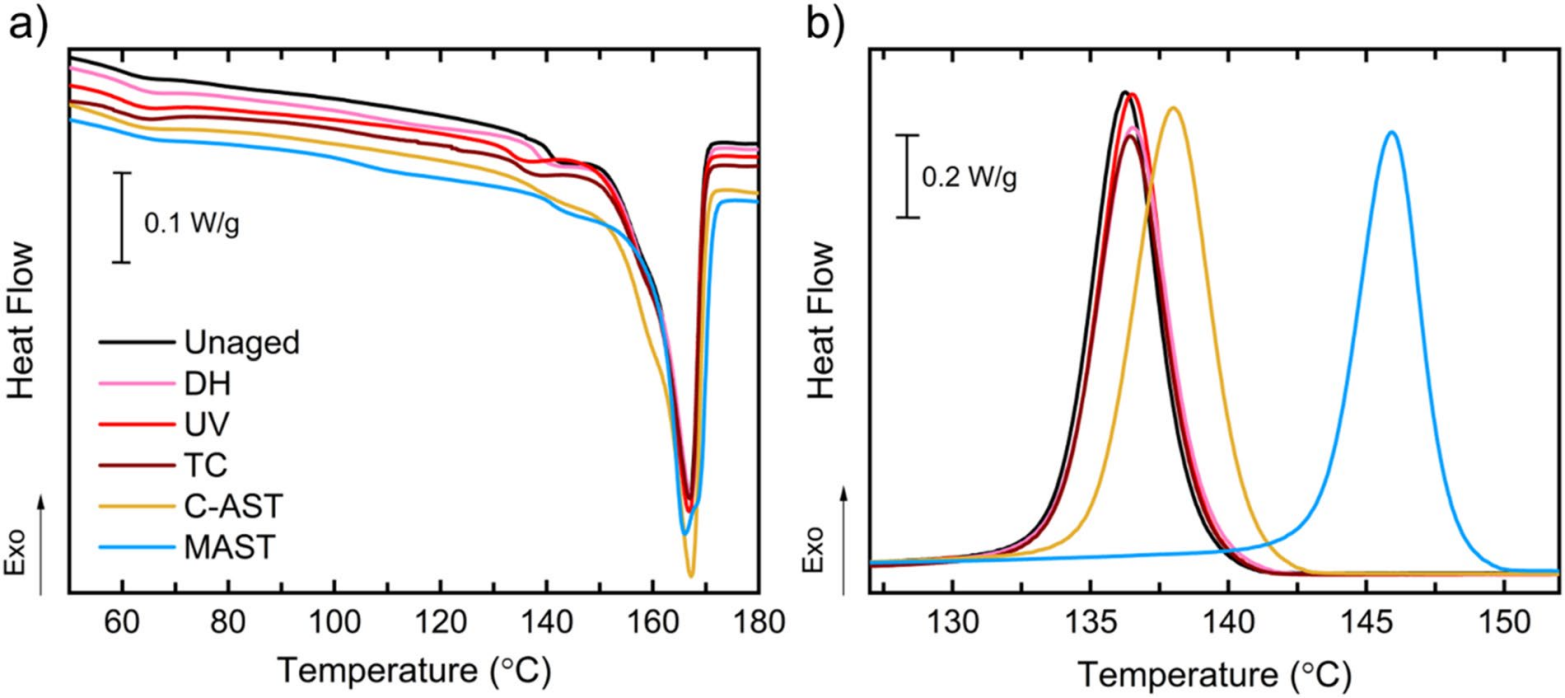
DSC thermograms of single-layer PVDF-A: (**a**) first heating cycle and (**b**) cooling cycle. The curves are offset vertically in (**a**) to better distinguish the experiments.

Figure 3 shows DSC thermograms of heating and cooling cycles for PVDF-A. The first DSC heating cycle was used to evaluate physical changes upon aging, such as degree of crystallinity and crystal perfection. In PVDF-A, the main melting peak was found at 167 °C for all test conditions. The degree of crystallinity was obtained by integration of the melting peak between 100 and 180 °C using a linear baseline, as explained in the following section, and the values are summarized in Supplementary Table S2 in the SI. Here, the PMMA is amorphous and does not contribute to the heat of fusion, therefore the qualitative changes in DSC thermograms are representative of the semi-crystalline PVDF. However, PMMA content affects the reported degree of crystallinity, as it contributes to the total weight of the specimen. Negligible changes in crystallinity in PVDF-A were observed for all test conditions except for C-AST, after which a small increase was measured. Previously, an increase in crystallinity after DH was reported due to PVDF post-crystallization during annealing at chamber temperatures above the polymer glass transition^23,29^.

The chain scission reactions that caused loss of PMMA are evidenced in the cooling DSC cycle. In particular, the crystallization peak shifted to higher temperatures—by 10 °C for MAST, 1.5 °C for C-AST, and, less than 0.3 °C for all the single-stress test conditions. Similar DSC peak shifts have previously been observed with varying PVDF/PMMA ratios^25^. Due to suspected mass loss from PMMA depletion, DSC is inconclusive on the changes in crystallinity after MAST aging. WAXS diffractograms are useful for qualitatively assessing changes in crystallinity. WAXS of PVDF-A (Supplementary Fig. S2 in the SI) confirms an increased growth of the α-PVDF in the (020) plane after the MAST sequence. Supplementary Fig. S2 shows that after MAST, the preferred orientation of the backsheet changed from (110) plane to the (020) plane of the α-PVDF, which was not observed for any other sample. We hypothesize that annealing the polymer during the high temperature MAST test sequences together with chemical damage including chain scission and PMMA depletion evidenced from DSC and FTIR resulted in conditions which favored PVDF orientation along the (020) crystal plane rather than along the (110) plane. All single-stress test conditions show negligible changes in the DSC thermograms, indicating minimal physical or permanent chemical damage to the backsheet.

Mechanical properties are readily influenced by polymer characteristics such as crystallinity, morphology, and structure. PMMA has greater elastic modulus compared to PVDF^3^, and the loss of PMMA, breaking of the polymeric chain, and increase in crystallinity during MAST and C-AST aging likely reduced the critical strain to fracture of the backsheet outer layer. This is consistent with the observation of crack formation in the machine direction on PVDF-A after MAST and C-AST protocols, following the loss in PMMA^15,19^, but not after any of the single-stress test conditions, where PMMA content was retained.

### Changes in crystallinity (PVDF-B)

**Figure 4:**
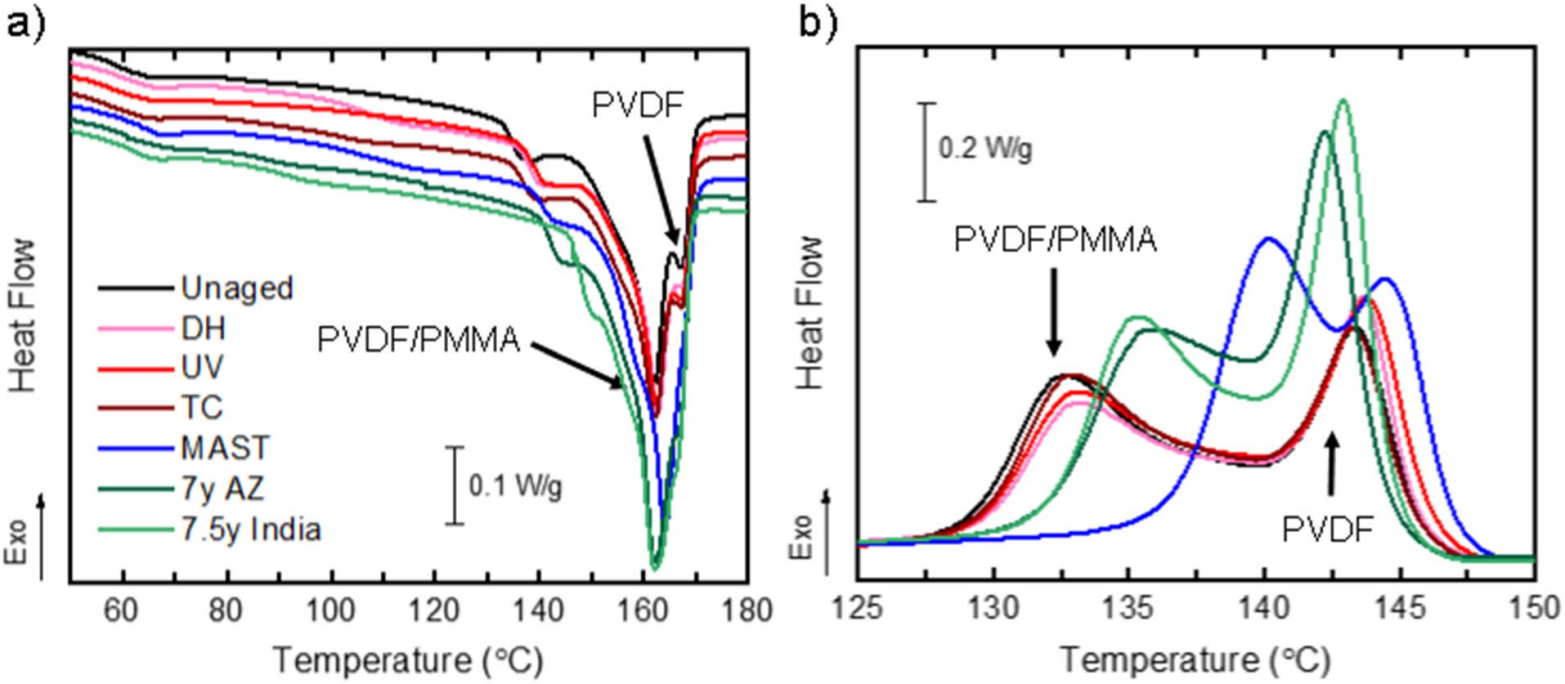
DSC thermograms of three-layer PVDF-B: (**a**) first heating and (**b**) cooling cycles comparing single (DH, UV, TC), sequential (MAST), and outdoor weathering with the unaged backsheet specimen. The arrows indicate the contributions of the distinct layers of PVDF-B. The curves are offset vertically in (**a**) to better distinguish the experiments.

The three-layer PVDF-B has a thin layer (~ 5 μm) of PVDF on the surface, which protects the PVDF/PMMA blend from direct exposure, as PVDF has excellent UV and thermal resistance^2^. Figure 4 shows DSC of the first heating and cooling cycles of the PVDF-B. The unaged film shows two distinct PVDF melting peaks, with the main peak at 162 °C and a second, lower intensity peak at 167 °C. The cooling cycle also shows the presence of two distinct crystallization peaks, at 133 °C and 143.5 °C. These two peaks are attributed to the melting and crystallization of the two distinct layers, the core PVDF/PMMA blend (T_m_ = 162 °C, T_c_ = 133 °C) and the outer/inner PVDF (T_m_ = 167 °C, T_c_ = 143.5 °C), where T_m_ and T_c_ are melting and crystallization temperatures, respectively. Integrating the area under the melting and crystallization peaks determines the enthalpy of fusion (ΔH_m_) and crystallization (ΔH_c_) of PVDF-B. The enthalpy of fusion is used to calculate the degree of crystallinity (χ_C_) of the polymer blend using Eq. (1), where $\Delta {H}_{m}^{0}$ is the theoretical enthalpy of fusion of 100% crystalline PVDF polymer, 104.7 J/g^30^.

1 $${\chi }_{C}\left(\%\right)=\frac{\Delta {H}_{m}}{\Delta {H}_{m}^{0}}\times 100 \%.$$

Table 2 summarizes the values of temperature transitions and enthalpies extracted from the DSC first heating and cooling cycles, as well as the calculated degrees of crystallinity for the various test conditions. From the cooling cycle, minor shifts (< 1 °C) of both crystallization peaks to higher temperatures were observed for single-stress test conditions. In addition, only small changes to the degree of crystallinity were measured, and these changes could partially result from uncertainty in the measurement or integration limits. Out of the single-stress test conditions, DH caused the most significant increase in crystallinity, which is consistent with previous studies and suggests that this test may not be appropriate for replicating field-relevant degradation^29^. There was no evidence of cracking of the backsheet layer after aging with these single-stress tests; however, no mechanical load was applied. PVDF-B backsheets cracked in MAST and in the field^15,31^. Furthermore, the MAST-aged PVDF-B was extremely brittle and shattered upon touching. Similar to MAST-aged PVDF-A, the PVDF/PMMA crystallization peak in PVDF-B shifted to higher temperatures by 7.5 °C after MAST, which is indicative of depletion of the PMMA polymer. The PMMA depletion occurred despite the fact that the PVDF/PMMA layer was protected by an unblended PVDF layer and therefore was not directly exposed to the chamber environment. For the fielded backsheets, PMMA depletion was also evident, but to a lesser extent than for MAST (crystallization peak shifts of 2.7 °C and 3.3 °C were observed for India and Arizona, respectively).

**Table 2:** Summary of parameters extracted from DSC first heating and cooling cycles for PVDF-B.

		T_m_ (°C) First heating	ΔH_m_ (J/s) First heating	T_c_ (°C) Cooling	ΔH_c_ (J/s) Cooling	χ_C_ (%)
Unaged		161.8	34.3	143.4	31.2	32.8
Single	DH	162.4	36.9	143.7	32.3	35.2
	UV	162.3	33.7	143.7	30.6	32.2
	TC	162.1	33.3	143.2	31.1	31.8
Sequential	MAST	163.9	39.3	140.2	34.5	37.5
Fielded	7 years, Arizona	162.1	43.4	142.3	38.7	41.5
	7.5 years, India	162.1	42.4	143.0	39.8	40.5

**Figure 5:**
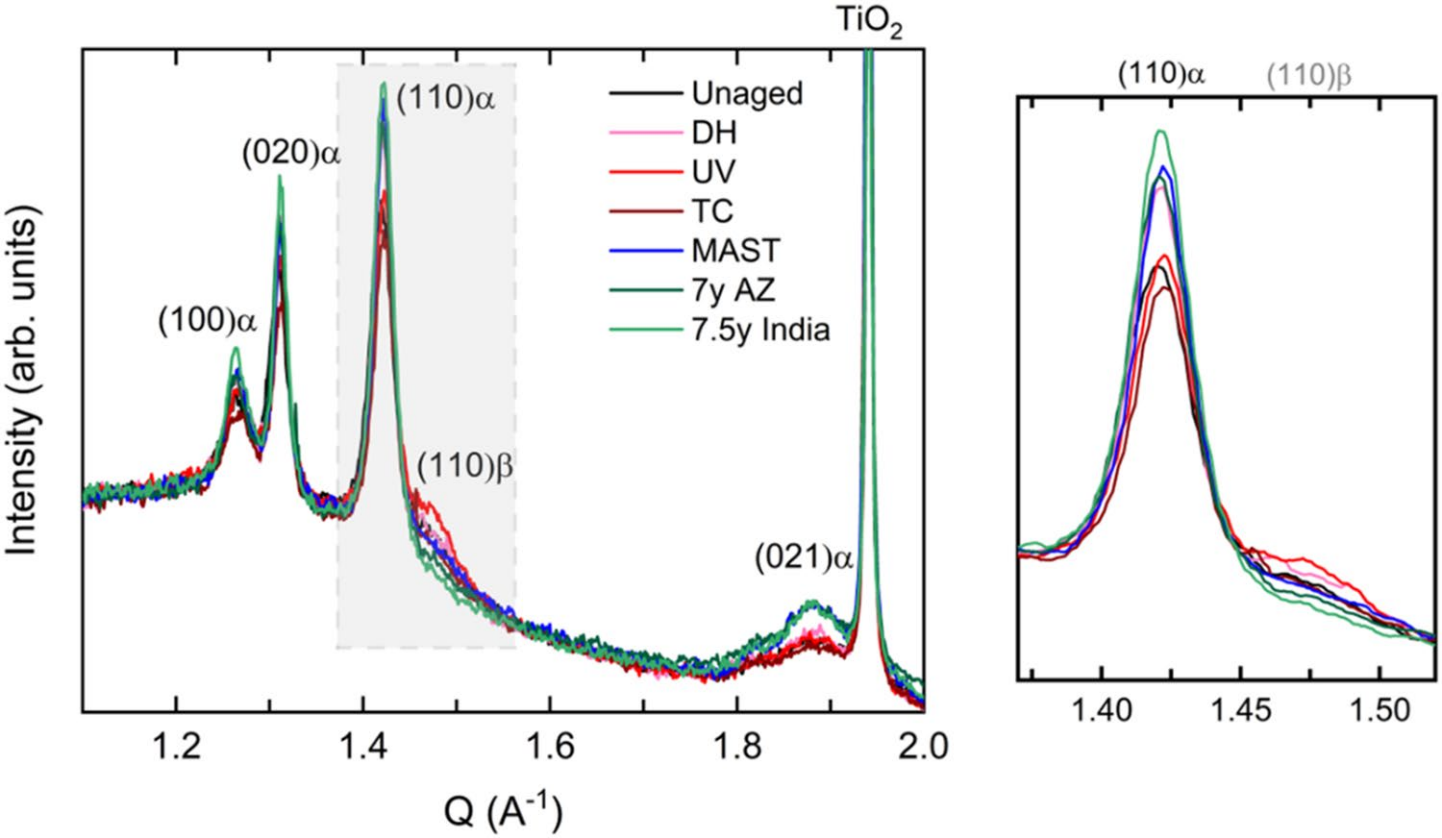
WAXS diffractograms identifying crystal planes corresponding to PVDF α- and β-phases and TiO_2_ pigment in PVDF-B aged with different test conditions. The inset on the right shows the detail of the peaks corresponding to (110)α and (110)β crystal phases compared in Table 4.

The degree of crystallinity increased by nearly twofold for the Arizona-aged sample compared to MAST. However, as noted above, the expressed values do not consider PMMA (or PVDF) mass loss, which would affect the degree of crystallinity, as both contribute to the total weight of the sample but only PVDF is crystalline. This may have resulted in an under/overestimation of the degree of crystallinity, in particular for the MAST-aged sample, where the most significant DSC peak shifts and intensity changes occurred, suggesting acrylic loss or outer layer erosion. Corresponding WAXS data (Fig. 5) is discussed in more detail in the following section, but the relative peak intensities show the greatest increase in crystallinity for the fielded PVDF-B from India, followed by MAST, Arizona, and DH, respectively. UV showed no change, and TC showed a small reduction in the crystallinity of the PVDF α-phase.

The increase in crystallinity in fielded samples is from crystallization of the PVDF α-phase (Fig. 5), mainly from the unblended PVDF outer layer of PVDF-B, as indicated by the increase of corresponding peaks in DSC (Fig. 4). This increase is less noticeable in the MAST-aged sample and can be an indication of surface erosion. The PVDF crystallization peak shifts to higher temperatures are seen after UV and DH (by 0.4 °C) and MAST (by 1.1 °C). These suggest minor degradation of the outer PVDF by chain scission reactions. On the other hand, the PVDF crystallization peak shifted by − 0.5 and − 1.1 °C for India and Arizona backsheets, respectively. This negative shift can be related to a number of factors, including phase transformation, morphological changes, additive depletion, or cross-linking. These results emphasize that the complexity of the outdoor environment is extremely hard to replicate, even using multi-stress accelerated tests.

To evaluate changes in backsheet crystallinity after C-AST aging, DSC of C-AST-aged PVDF-A is shown in Supplementary Fig. S3a in the SI. Table 3 summarizes the changes in DSC parameters for PVDF-A subjected to C-AST as compared to MAST (discussed in the previous section). Cracks in the machine direction over outward-facing topographic features started to appear after 3 months of C-AST aging. Cracks rapidly propagated over the entire backsheet with further C-AST aging. The backsheet properties were analyzed at 3 and 6 month read points. The DSC crystallization peak shifted to higher temperatures by 1.5 °C after 3 months and a further 0.2 °C after an additional 3 months of weathering. The degree of crystallinity increased by 17% after 3 months; however, a small decrease was observed after 6 months (8% of increase compared to unaged). Similar to MAST, this value may be affected by PMMA depletion and surface erosion, if these took place and continued during further aging. Supplementary Fig. S3b in the SI shows FTIR spectra from the surface of C-AST-aged backsheets after 3 and 6 months. The PMMA carbonyl peak at 1730 cm^−1^ was reduced only minimally after 3 months of aging; however, further aging seemed to promote PMMA depletion from the single-layer PVDF-PMMA blend. The minimal DSC crystallization peak shift (0.2 °C) between 3 and 6 months of C-AST suggests PMMA depletion mainly from the surface of the backsheet.

**Table 3:** Summary of DSC parameters for PVDF-A after C-AST and MAST Aging.

	T_m_ (°C) First heating	ΔH_m_ (J/s) First heating	T_c_ (°C) Cooling	ΔH_c_ (J/s) Cooling	χ_c_ (%)
Unaged	166.9	25.6	136.3	25.8	24.5
MAST	166.1	25.3	145.9	24.3	24.2
C-AST 3 months	167.3	30.1	137.8	27.2	28.7
C-AST 6 months	167.3	27.6	138.0	27.6	26.4

Although PMMA depletion from the backsheet layer is undesirable because it can leave behind microscopic pores and voids that could initiate crack formation^28^, the increase in crystallinity observed in fielded samples as well as after MAST and C-AST is likely the primary driver of backsheet cracking. Although no mechanical tests were performed in this work, several studies have observed embrittlement of the PVDF material after DH from post-crystallization, making the samples un-measurable^23,29^. An increase in crystallinity has been correlated with a decrease in tear energy and tensile properties from polymer embrittlement in high-density polyethylene (HDPE) and PET used in backsheets^32,33^. In addition, the PVDF α to β crystalline phase transformation has been identified as a potential factor in backsheet cracking, as it may also induce changes in mechanical properties^16,34^.

In terms of the applied stress, neither single-stress test conditions nor the MAST protocol seemed to closely replicate the effects of the natural environment on PVDF-based backsheets. On the one hand, single-stress test conditions such as DH, UV, and TC alone were not sufficient to produce the chemical degradation and/or increase in crystallinity that would induce crack formation and backsheet failure. On the other hand, while MAST did produce cracks on PVDF-based backsheets, the test sequence applied here may have been too harsh, causing excessive degradation. Although the MAST test used here is extreme, it allows the identification of weaknesses in PVDF-based backsheets that single-stress test conditions overlook. Although C-AST cannot be directly compared to the outdoor data, C-AST protocols were successful in inducing changes in material characteristics (i.e., increase in crystallinity, PMMA degradation), leading to crack formation without being as extreme as MAST.

### Impact of the PVDF β-phase

Although the PVDF α-phase is the most commonly seen phase in PVDF-based backsheets, PVDF can undergo a structural phase transformation under applied stress. The α- to β-phase transformation can take place via mechanical deformation (stretching) of the α-phase. The exact location of the β-phase formation, its effects on material properties, and its relation to backsheet cracking are subjects of ongoing research. The two major phases, α and β, can be distinguished by WAXS and FTIR based on the position of their characteristic scattering peaks and vibrational bands, respectively^26,35^. WAXS diffractograms of PVDF-B (Fig. 5) show the presence of sharp peaks corresponding to PVDF α-phase at Q = 1.27 Å^−1^ (100), 1.31 Å^−1^ (020), 1.42 Å^−1^ (110), and 1.88 Å^−1^ (021). The peak at Q = 1.94 Å^−1^ corresponds to TiO_2_ pigment. The shoulder present at Q = 1.47 Å^−1^ can be identified as the reflection of (110) β-phase^35^. As discussed earlier, the intensity increase of peaks corresponding to the PVDF α-phase is apparent for fielded, MAST-aged, and DH-aged samples. The amplitude ratio of (110)β and (110)α peaks is expressed in Table 4. A small amount of β-phase was detected in all samples, including the unaged sample, which indicates that some β-phase was formed during the backsheet fabrication process. Relative to the unaged specimen, the proportion of the β-phase identified by WAXS seemed to increase the most after DH, UV, and TC, but dropped after outdoor weathering in India and Arizona. No evidence of the presence of PVDF β-phase from WAXS was seen before or after aging in PVDF-A type backsheets (see Supplementary Fig. S2 in the SI).

**Table 4:** Relative peak intensity ratio of PVDF β- to α-phase from two different phase identification methods, WAXS and FTIR, in PVDF-B backsheets aged with different test conditions.

		WAXS (110)β/(110)α	FTIR β (1275 cm^−1^)/α (1180 cm^−1^)
Unaged		0.095 ± 0.040	0.110 ± 0.027
Single	DH	0.137 ± 0.028	0.136 ± 0.020
	UV	0.189 ± 0.039	0.083 ± 0.050
	TC	0.151 ± 0.040	0.052 ± 0.016
Sequential	MAST	0.094 ± 0.029	0.113 ± 0.018
Fielded	7 years, Arizona	0.072 ± 0.038	0.087 ± 0.075
	7.5 years, India	0.049 ± 0.032	0.117 ± 0.035

**Figure 6:**
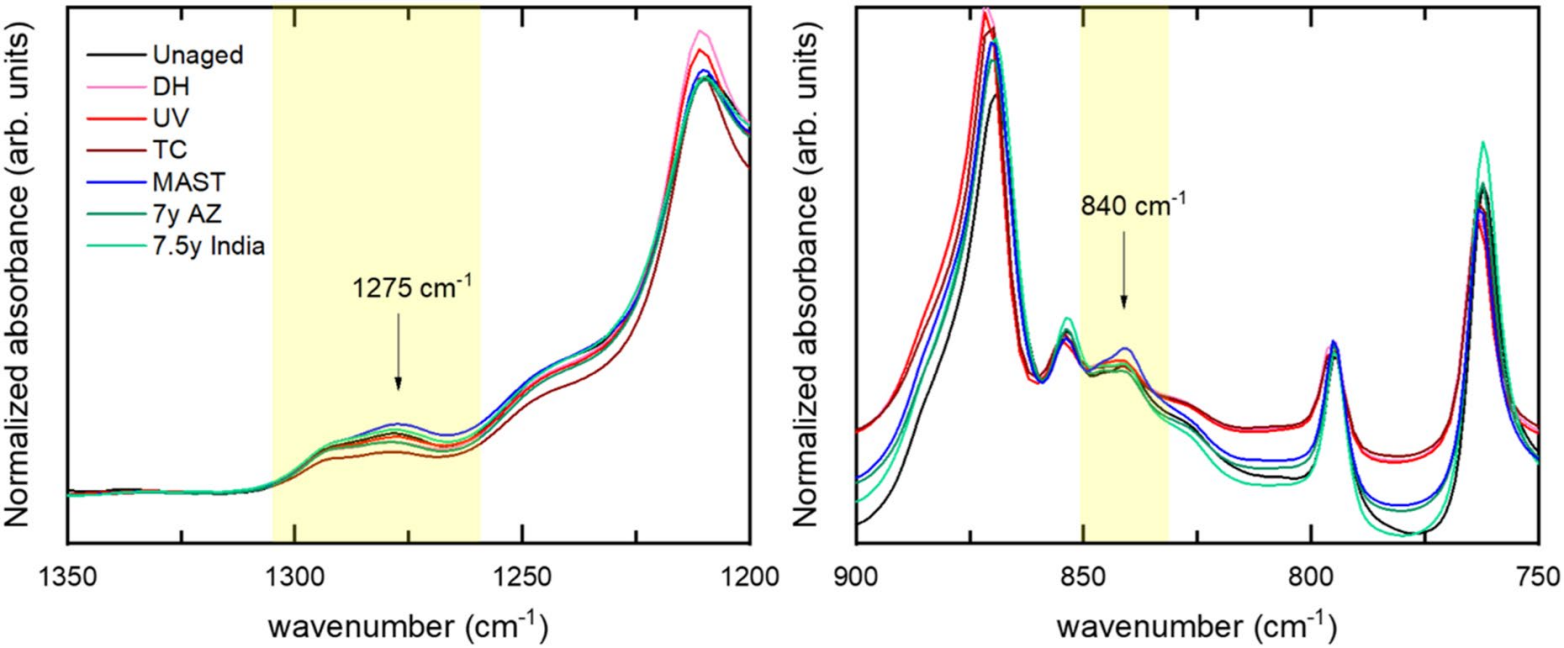
FTIR spectra of PVDF-B, focusing on the regions of spectra specific to PVDF β-phase.

As for PVDF-A, the FTIR spectra of PVDF-B (Supplementary Fig. S1 in the SI) confirm that the backsheet is predominantly PVDF α-phase. Figure 6 shows two regions of the PVDF-B spectra, focusing on the β-phase- specific vibrations found at 840 cm^−1^ and 1275 cm^−1^^26^. The intensity ratio between the peaks at 1275 cm^−1^ (β-phase) and 1180 cm^−1^ (α-phase) is summarized in Table 4. FTIR suggests that the largest increase in the β-phase formation occurs after DH weathering, and a small increase occurs after MAST and outdoor weathering in India, but a decrease is seen after UV and TC. It is clear that the trends in relative quantification of the β-phase from WAXS do not correspond to those from FTIR. In addition, WAXS does not suggest the presence of β-phase in any of the PVDF-A samples, whereas FTIR shows the presence of β-phase and its increase after C-AST, MAST, and TC (Fig. 2). A small amount of β-phase formed locally on the studied PVDF backsheets was also observed by Weiser et al. when performing multiple FTIR measurements^34^.

The differences in identification and quantification of the PVDF β-phase from WAXS and FTIR spectra may be related to differences in how the two techniques interact with the sample as well as the location and depth of the measurements. In FTIR, the infrared light absorbed by the sample depends on the presence of specific covalent bonds. FTIR collects data from an area of 1.8 mm—defined by the crystal used—and it is a surface-sensitive technique with depth penetration of approximately 2 μm. Although WAXS also probes the sample on the atomic scale, the measurement is done on a 100 μm diameter spot and throughout the entire backsheet layer thickness. Furthermore, UV damage is typically only present within a few micrometers of the surface, and therefore FTIR may be more sensitive to this type of degradation. In contrast, thermal degradation affects the entire backsheet thickness, and thus WAXS is better at identifying it. Previous studies have suggested that β-phase may form locally in regions with large deformation, such as in proximity of a crack tip^36^. Further investigation of this region is necessary to draw conclusions about the formation of β-phase and its relation to backsheet failure. This work indicates that a small amount of β-phase may coexist with α-phase locally, but there is no evidence of excessive β-phase formation after outdoor or accelerated weathering.

## Conclusions

This work focuses on understanding the changes in material properties that lead to mechanical failure of PVDF-based backsheets in the field. PVDF chemical degradation, changes in polymer degree of crystallinity, and phase transformation are discussed. The PVDF-based backsheets studied here underwent various single (UV, DH, TC), sequential (MAST), and combined (C-AST) accelerated stress tests with the goal of finding the most suitable test to replicate premature backsheet degradation in the field.

The complexity of the PVDF-based layer in backsheets arises from the fact that its composition (PMMA content, additives, and pigments), structure, and fabrication process will affect its chemistry, crystallography, and corresponding mechanical properties. This work showed that combined or sequential aging can lead to PMMA loss from the backsheet, regardless of whether the PMMA is exposed to the environment or protected by a PVDF matrix. DSC and WAXS techniques identified a significant increase in crystallinity in fielded backsheets from Arizona and India, and the increase in crystallinity in PVDF-based backsheets was also reproduced to some extent with MAST and C-AST aging. The changes in crystallinity occurred through an increase of PVDF α-phase, which is the dominant phase in the studied backsheets. FTIR and WAXS confirmed the local presence of the PVDF β-phase; however, the exact location of β-phase formation and its correlation with specific test conditions are inconclusive. No excessive β-phase formation was observed after the field or accelerated tests, suggesting that it was the overall increase in crystallinity that caused polymer embrittlement and consequent cracking in the studied samples. Further investigation of the local β-phase formation and its relation to cracking is ongoing.

Single-stress test conditions (UV, DH, TC) alone did not show evidence of PMMA depletion or a significant increase in PVDF crystallinity, corresponding to the absence of backsheet failure in the form of cracking. Of the studied tests, the MAST sequence was the most extreme, producing excessive PVDF degradation. Neither of the single or sequential tests closely replicated the degradation mechanisms seen in the fielded backsheets; however, the MAST test was useful in identifying weaknesses in the material that led to failure. Although C-AST cannot be directly compared to the fielded samples, it was successful in replicating PVDF backsheet cracking during the “desert” sequence, while limiting the excessive PMMA degradation observed in MAST. Despite the fact that C-AST and MAST tests did not completely replicate the complex nature of the outdoor environment, both tests are valid for screening outdoor failure mechanisms in PVDF backsheets.

## Supplementary Information

Supplementary Information.

## Data Availability

The datasets generated and/or analyzed during the current study are available in the DuraMAT Datahub, https://datahub.duramat.org/.
