# Supplementary material for: A study of degradation mechanisms in PVDF-based photovoltaic backsheets
Source: Sci Rep. 2022 Aug 24;12:14399. doi: 10.1038/s41598-022-18477-1 (PMC9402549; doi:10.1038/s41598-022-18477-1)
Supplement: Supplementary file 1 — Supplementary Information. [file 41598_2022_18477_MOESM1_ESM.pdf]

Supporting Information

A study of degradation mechanisms in PVDF-based photovoltaic backsheets

Soňa Uličná[1], Michael Owen-Bellini[2], Stephanie L. Moffitt[1], Archana Sinha[1], Jared Tracy[3], Kaushik Roy-Choudhury[3], David C. Miller[2], Peter Hacke[2], and Laura T. Schelhas[2]

[1] SLAC National Accelerator Laboratory, Menlo Park, CA, USA

[2] National Renewable Energy Laboratory, Golden, CO, USA

[3]DuPont Specialty Products LLC, Wilmington, DE, USA

Table S1: Summary of Accelerated and Outdoor Stress Test Conditions Applied

<table>
<tr><th>Form</th><th colspan="3">Test</th><th>DH</th><th>UV</th><th>TC</th><th>Freeze & Mechanical Load</th></tr>
<tr><td rowspan="4">Coupon</td><td>Unaged</td><td colspan="2"></td><td></td><td></td><td></td><td></td></tr>
<tr><td rowspan="3">Single</td><td colspan="2">Damp Heat (DH)</td><td>1,000 h,
85 °C, 85 % RH</td><td></td><td></td><td></td></tr>
<tr><td colspan="2">UV</td><td></td><td>1,500 h,
65 W/m² at 340 nm, 65 °C</td><td></td><td></td></tr>
<tr><td colspan="2">Thermal Cycling (TC)</td><td></td><td></td><td>200 h,
-40 °C to 85 °C</td><td></td></tr>
<tr><td rowspan="3">Mini-Module</td><td>Sequential</td><td colspan="2">MAST</td><td>1,000 h,
85 °C, 85 % RH</td><td>4x 1,000 h,
65 W/m² at 300-400 nm, 70 °C</td><td>3x 200 h,
-40 °C to 85 °C</td><td></td></tr>
<tr><td rowspan="2">Combined</td><td rowspan="2">C-AST</td><td>Tropical</td><td>40 °C,
95 % RH,
Rain Spray</td><td>1.9 Suns*,
90 °C, 28 % RH
Sys. Voltage</td><td></td><td>-20 °C,
28 %/95 % RH,
Mech. load</td></tr>
<tr><td>Multi-Season</td><td></td><td colspan="2">0.8 Suns*,
0 °C, 6 % RH
Sys. Voltage
&
1.9 Suns*,
90 °C, 6 % RH</td><td>-40 °C,
20 % RH,
Mech. load</td></tr>
<tr><td rowspan="2">Module</td><td rowspan="2">Fielded</td><td colspan="2">7 years, Arizona</td><td colspan="4"></td></tr>
<tr><td colspan="2">7.5 years, India</td><td colspan="4"></td></tr>
</table>

* Suns as indicated is filtered Xenon solar simulation incident on the module front with 8% albedo arriving on the rear (backsheet) by means of reflectors

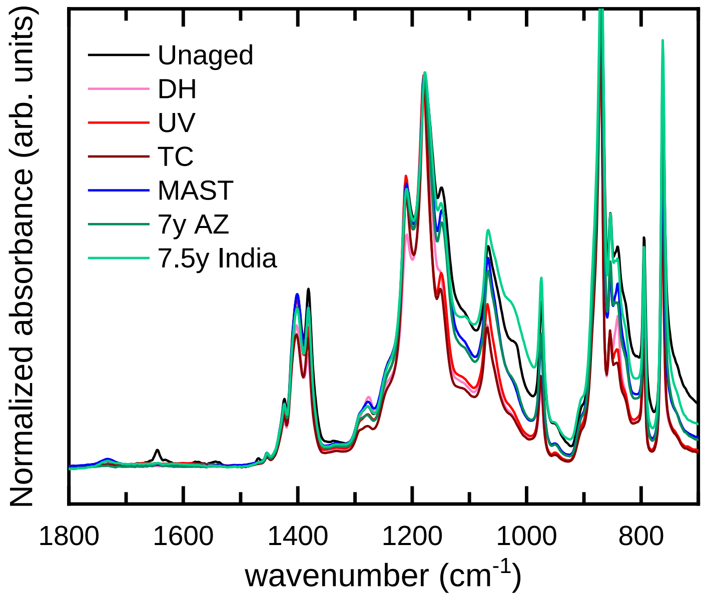

Figure S1: Normalized FTIR spectra of the weathering-exposed surface of the three-layer PVDF-B.

Table S2: Summary of DSC Parameters for PVDF-A

	T_m (°C) First Heating	ΔH_m (J/s) First Heating	T_c (°C) Cooling	ΔH_c (J/s) Cooling	χ_c (%)
**Unaged**	166.9	25.6	136.3	25.8	24.5
**DH**	167.2	25.6	136.6	25.9	24.5
**UV**	166.9	24.1	136.5	26.7	23.0
**TC**	167.1	24.2	136.5	24.4	23.1
**MAST**	166.1	25.3	145.9	24.3	24.2
**C-AST 3 months**	167.3	30.1	137.8	27.2	28.7
**C-AST 6 months**	167.3	27.6	138.0	27.6	26.4

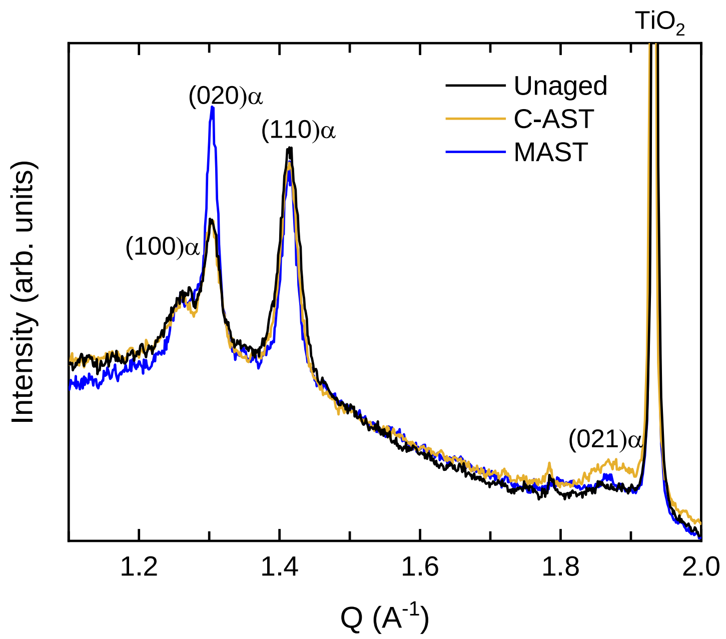

Figure S2: WAXS diffractograms identifying only PVDF α-phase and TiO_2 pigment in PVDF-A aged with C-AST and MAST. C-AST data shown here is for 3 months of aging. Negligible changes were observed between 3 and 6 months of C-AST aging.

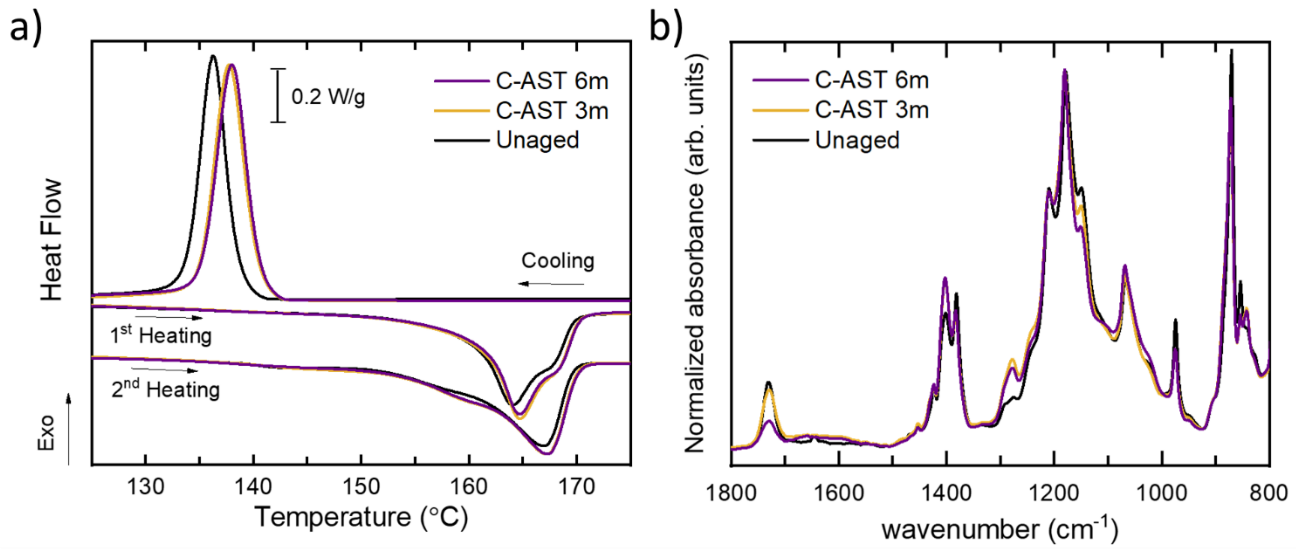

Figure S3: (a) DSC thermograms and (b) FTIR spectra of PVDF-A before and after C-AST aging for 3 and 6 months.
